# Mathematical model combined with microdosimetric kinetic model for tumor volume calculation in stereotactic body radiation therapy

**DOI:** 10.1038/s41598-023-38232-4

**Published:** 2023-07-06

**Authors:** Hisashi Nakano, Takehiro Shiinoki, Satoshi Tanabe, Satoru Utsunomiya, Takeshi Takizawa, Motoki Kaidu, Teiji Nishio, Hiroyuki Ishikawa

**Affiliations:** 1 Department of Radiation Oncology, Niigata University Medical and Dental Hospital, 1-757 Asahimachi-dori, Chuo-ku, Niigata-shi, Niigata Japan; 2 Department of Medical Physics and Engineering, Osaka University Graduate School of Medicine, 1-7 Yamadaoka, Suita-shi, Osaka, Japan; 3 Department of Radiation Oncology, Yamaguchi University, Minamikogushi 1-1-1 Ube, Yamaguchi, Japan; 4 Department of Radiological Technology, Niigata University Graduate School of Health Sciences, 2-746 Asahimachi-Dori, Chuo-ku, Niigata-shi, Niigata Japan; 5 Department of Radiation Oncology, Niigata Neurosurgical Hospital, 3057 Yamada, Nishi-ku, Niigata-shi, Niigata Japan; 6 Department of Radiology and Radiation Oncology, Niigata University Graduate School of Medical and Dental Sciences, 1-757 Asahimachi-dori, Chuo-ku, Niigata-shi, Niigata Japan

**Keywords:** Computational science, Cancer models, Radiotherapy, Non-small-cell lung cancer

## Abstract

We proposed a new mathematical model that combines an ordinary differential equation (ODE) and microdosimetric kinetic model (MKM) to predict the tumor-cell lethal effect of Stereotactic body radiation therapy (SBRT) applied to non-small cell lung cancer (NSCLC). The tumor growth volume was calculated by the ODE in the multi-component mathematical model (MCM) for the cell lines NSCLC A549 and NCI-H460 (H460). The prescription doses 48 Gy/4 fr and 54 Gy/3 fr were used in the SBRT, and the effect of the SBRT on tumor cells was evaluated by the MKM. We also evaluated the effects of (1) linear quadratic model (LQM) and the MKM, (2) varying the ratio of active and quiescent tumors for the total tumor volume, and (3) the length of the dose-delivery time per fractionated dose (t_inter_) on the initial tumor volume. We used the ratio of the tumor volume at 1 day after the end of irradiation to the tumor volume before irradiation to define the radiation effectiveness value (REV). The combination of MKM and MCM significantly reduced REV at 48 Gy/4 fr compared to the combination of LQM and MCM. The ratio of active tumors and the prolonging of t_inter_ affected the decrease in the REV for A549 and H460 cells. We evaluated the tumor volume considering a large fractionated dose and the dose-delivery time by combining the MKM with a mathematical model of tumor growth using an ODE in lung SBRT for NSCLC A549 and H460 cells.

## Introduction

Stereotactic body radiation therapy (SBRT), which is widely used in the treatment of early-stage non-small cell lung cancer (NSCLC), is characterized by delivering high doses with a small number of divisions^1–3^. A rapid dose reduction from the target and optimal target dose compatibility are critical to minimizing toxicity to normal tissue when SBRT is administered^4,5^. In a comparison of the outcomes of SBRT and surgery, there was no significant difference in the percentage of patients who were alive at 5 years after treatment: 87% for SBRT versus 84% for surgery. There was also no significant difference in the percentage of patients who were alive without recurrence at 5 years after treatment: 77% for SBRT versus 80% for surgery. Severe complications associated with treatment were less common with SBRT (about 1%) than with surgery^6^. There were no instances of 90 days mortality (0%), the bleeding requiring re-admission (1/80, 1.3%), and one of 80 patients (1.3%) required postoperative admission to the intensive care unit. Various other clinical trials of SBRT have been conducted and reported results that were comparable to those of surgery^7,8^.

Mathematical models have been used to evaluate the complex responses in human physiological and pathological processes and have been extended to many areas^9,10^. Mathematical models were used to calculate the various reactions in medicine, vaccine efficacy, and for predicting the effects of anticancer drugs^11–13^. An ordinary differential equation (ODE) in mathematical models is a set of differential equations containing an independent variable and one or more derivatives for that variable. An ODE is the most extensive form for modeling dynamical systems in science and engineering^10,14,15^. In systems biology, many biological processes (e.g., gene regulation and signal transduction) can be modeled by reaction rate equations that express the rate of the production of one species as a function of the concentration of another species in the system^11,13,14,16,17^. Evaluations of tumor growth using an ODE-based mathematical model in radiation therapy of tumors with single irradiation have been also been reported^18^. With the use of a mathematical model based on an ODE, it becomes possible to evaluate the tumor volume as an output, determine the effect of radiation therapy on the tumor, and derive the optimal irradiation schedule, irradiation dose, and the tumor effect when radiation therapy is combined with drugs^19–21^.

In order to compare the biological effects of radiotherapy administered with different doses and differing numbers of radiation treatments, mainly the following equation, called the linear quadratic model (LQM), is used in current radiotherapy^22,23^. Comparisons of the effects of the LQM have been made with single doses in the range commonly used in radiotherapy up to approx. 8 Gy^24,25^. In the LQM, the surviving fraction (SF) is calculated as a function of the absorbed dose (D) in Gy with two coefficients, α and β, where α is the proportionality factor to D [Gy^−1^ ] and β is the proportionality factor to D^2^ [Gy^−2^]. However, the single fractionated dose exceeds 10 Gy in SBRT applied to lung cancers, and the actual cell survival rate may be higher than that predicted by the LQM when the dose is > 10 Gy^26–29^.

Some reports suggest that the LQM does not agree with the measured SF at high doses because the LQ curve bends continuously on a log-linear plot, which may interfere with extrapolation to high-dose fractionated treatments^28,30,31^. Various cell survival models have been proposed to solve this problem, microdosimetric kinetic model (MKM) was proposed that can predict the cell SF from physical doses based on domains, which are intracellular structures, for all types of radiation^32^. An MKM can accurately calculate cell viability even in the high-dose range by taking into account the radiation quality, the dose rate, and the cell DNA repair time^33–37^. There is a possibility of overestimating the effect of SF in the evaluation of LQMs, which may have a higher predictive at a single large dose since the calculation of cellular SF during irradiation by mathematical models based on ODEs is evaluated using LQM^19–21^. In addition, the LQM cannot account for sublethal damage repair (SLDR) affecting a tumor’s survival during irradiation, and there are few reports of the effect of the dose-delivery time used to irradiate a prescribed dose on the tumor cell volume in mathematical models based on ODEs.

We therefore created a new mathematical model by combining an ODE and an MKM, and we describe the model below. Based on our validation of the model, we propose that this combination model can be used to predict the tumor cell lethal effect of SBRT administered to NSCLC.

## Methods

### The SF calculations for 6MV photon beams using the MKM

The cell nucleus is divided into hundreds of independent regions, which are called domains in an MKM^32^. Irradiation of these domains causes a potential lethal lesion (PLL). PLLs are classified into the following four categories according to their variants. (1) Irreparable lethal lesions (LLs) that appear in the primary process (‘a’ is the conversion rate constant); (2) PLLs that are converted to LLs in the secondary process (‘b_d_’ is the conversion rate constant); (3) lesions that can be repaired in the primary process (‘c’ is the repair constant; and (4) lesions that do not become LLs for a certain period of time (t_r_) and then become LLs and cannot be repaired.

The MKM assumes that a PLL is a double-strand break in DNA. The number of PLLs were caused a single instantaneous irradiation. The number of PLLs per domain using the rate constants of conversion (a, b_d_, c) for transformations is calculated as:

1 $$ \frac{dP}{{dt}} = - \left( {a + c} \right)P - 2b_{d} P^{2} $$

2 $$ \left( {a + c} \right) = \frac{\ln 2}{{T_{1/2} }} $$

3 $$ P = k_{d} ze^{{ - \left( {a + c} \right)t}} $$

Here, P is the number of PLLs in the domain and k_d_ is the average number of PLLs per domain per dose [Gy^−1^] immediately after the irradiation. The parameter ‘z’ is the specific energy stored in the domain [Gy], and ‘t’ is the time [h] after irradiation, satisfying 0 < t < t_r_. The t_r_ is assumed to be infinite, as discussed by Hawkins^32^. The repair rate (a + c) of the PLLs was equal to the primary repair rate λ calculated by the DNA repair half-life. The average number of LLs (L_n_) per cell nucleus ias defined as follows:

4 $$ \begin{aligned} L_{n} = & N\left\langle L \right\rangle = N\left\langle {A\left\langle z \right\rangle + Bz^{2} } \right\rangle \\ & = \left( {\alpha_{0 } + \gamma \beta_{0} } \right)D + \beta_{0} D_{2} \\ & = \left( {\alpha_{0} + \frac{{y_{D} }}{{\rho \pi r_{d}^{2} }}\beta_{0} } \right)D + \beta_{0} D^{2} \\ & = - l_{n} S \\ \end{aligned} $$

While the corresponding parameters are.

5 $$ \alpha_{0} = NA $$

6 $$ \beta_{0} = NB $$

7 $$ \gamma = \frac{{y_{D} }}{{\rho \pi r_{d}^{2} }} $$

Here, z is the specific energy [Gy] deposited in the domain, N is the number of domains, and A and B are coefficients. The parameter r_d_ is the radius of the domain (0.5 μm), ρ is the density of the domain (1.0 g/cm^3^), D is the absorbed dose (Gy), and y_D_ is the dose mean lineal energy (keV/μm). The parameters α_0_ and β_0_ were determined by a single instantaneous irradiation using the LQM.

The MKM has been improved to take into account various dose rates and irradiation schemes with photon beams and changes in the amount of DNA per nucleus during irradiation^38^. The irradiation time and irradiation interruption time were considered for the cell SF, and the changes in the amount of DNA in the cell cycle were ignored (α_0_ = constant, β_0_ = constant); by taking the limit and setting N to infinity, the expression in Eq. (4) is transformed as follows:

8 $$ \begin{aligned} \mathop {\lim }\limits_{N \to \infty } \left( { - l_{n} S} \right) = & \mathop {\lim }\limits_{N \to \infty } \mathop \sum \limits_{n = 1}^{N} \left[ {\left( {\alpha_{0} + \gamma \beta_{0} } \right)\dot{D}\Delta T + \beta_{0} \left( {\dot{D}\Delta T} \right)^{2} } \right] \\ & \quad + 2\mathop {\lim }\limits_{N \to \infty } \mathop \sum \limits_{n = 1}^{N - 1} \mathop \sum \limits_{m = n + 1}^{N} \left\{ {\beta_{0} \left[ {e^{{ - \left( {m - n} \right)\left( {a + c} \right)\Delta T}} } \right]} \right\}\left( {\dot{D}\Delta T} \right)^{2} \\ & = \left( {\alpha_{0} + \gamma \beta_{0} } \right)\dot{D}T + \beta_{0} \frac{2}{{\left( {a + c} \right)^{2} T^{2} }}\left[ {\left( {a + c} \right)T + e^{{ - \left( {a + c} \right)T}} - 1} \right] \dot{D}^{2} T^{2} \\ \end{aligned} $$

We thus transformed the Eq. (8) as follows:

9 $$ \begin{aligned} - l_{n} S = & \left( {\alpha_{0} + \gamma \beta_{0} } \right)D + F\beta_{0} D^{2} \\ & = \alpha_{MKM} D + \beta_{MKM} D^{2} \\ \end{aligned} $$

10 $$ D = \dot{D}T $$

Here, $\dot{\mathrm{D}}$ is the dose rate (Gy/min), and T is the dose-delivery time (min).

11 $$ F = \left\{ {\begin{array}{*{20}l} {\frac{2}{{\left( {a + c} \right)^{2} T^{2} }}\left[ {\left( {a + c} \right)T + e^{{ - \left( {a + c} \right)T}} - 1} \right] (T < t_{r} )} \hfill \\ {\frac{2}{{\left( {a + c} \right)^{2} T^{2} }}\left[ {\left( {a + c} \right)T - 1} \right] \left( {t_{r} \le T} \right)} \hfill \\ \end{array} } \right. $$

The parameter *F* is identified with the Lea–Catcheside time-factor G^39^. We used the α/β and DNA repair half-life T_1/2_ values of the parameters for calculating the DNA repair rate (a + c) value of two NSCLC cell lines, A549 and NCI-H460 (H460) to evaluate the radiation effect of SBRT^28,29,40–42^.

### The dose mean lineal energy y_D_ calculated by PHITS

The TrueBeam linear accelerator (Varian Medical Systems, Palo Alto, CA) using a 6MV X-ray beam was modeled with the particle and heavy ion transport code system (PHITS)^43^. PHITS can deal with photons, electrons, positrons, neutrons, and heavy ions^44^. The phase space files of the Monte Carlo (MC) that we applied were provided by Varian Medical Systems. The following phase space files were created using BEAMnrc built on the EGSnrc platform, and these phase space files created by BEAMnrc were transferred to the PHITS system in which the dose calculations were performed to simulate the 6MV photon beams. A water-equivalent phantom (20 × 20 × 20 cm^3^) was created; the beam field size was 5 × 5 cm^2^ with source to phantom surface distance (SSD) = 90 cm, and the measurement point was 10 cm deep. The calculation width was 3 cm in the water-equivalent phantom. The photon and electron cut-off energies were set to 0.01 MeV, and the MC calculation was performed with a statistical error < 1.0%. The dose-mean lineal energy y_D_ for MKM was calculated as:

12 $$ y = \frac{\varepsilon }{l} $$

13 $$ y_{D} = \frac{{\int {y^{2} } f\left( y \right)dy}}{{\int {yf} \left( y \right)dy}} = \frac{{\int {yd} \left( y \right)dy}}{\int d \left( y \right)dy} $$

where ε is the energy stored in the domain, l is the mean chord length, f(y) is the probability density of linear energy, and d(y) is the dose distribution of linear energy. The T-SED function of PHITS was used to calculate the dose mean lineal energy for 6 MV photon beams^45,46^. T-SED is a track structure T-SED calculates the distribution of energy imparted in a small area using formulas constructed based on the results of the analysis. The computationally derived y_D_ values were used to evaluate the cell survival of the tumor in Eq. (4).

### Tumor growth volume calculation using the ODE with mathematical model

We used the ODE with a multi-component mathematical model (MCM) that models growth by distinguishing the state and growth rate of tumor cells (active tumors and quiescent tumors, etc.) in this study^20^. The MCM distinguishes the tumor cells into active tumors, resting cells that can stop dividing and turn into active cells, and non-dividing cells, which are dead cells waiting to be excreted into the bloodstream. The effects on tumor cells were evaluated by combining the growth of tumor cells represented by the MCM with the mortality rate of cells expressed using the radiation calculation. The tumor cells were divided into the types T_1_, T_2_, and …Tm as active tumors in the MCM. The volume of the T_1_ tumor cells is V_1_; the volume of quiescent cells (T_Q_) is V_Q_, and the volume of the non-dividing cells (T_ND_) is V_ND_. The radiation affects active tumors (T_1_, T_2_,…T_m_) and quiescent cells (T_Q_), but not non-dividing cells (T_ND_). Because cells in the same tumor might be in different states and have different growth rates, such as active tumors and quiescent cells, the following model can be constructed using Eq. (14).

14 $$ \frac{{dV_{1} }}{dt} = a_{1} V_{1} \left( {1 - \frac{{V_{1} }}{{K_{1} }}} \right) + p_{Q1} V_{Q} - \left( {p_{1Q} + p_{1ND} } \right)V_{1} $$

The value of K_1_ is constant related to the tumor growth rate and the environmental carrying capacity. The parameter p_Q1_ represents the probability that a T_Q_ tumor cell is transformed into a T_1_ cell, and p_1Q_ is the probability that a T_1_ tumor cell is transformed into a T_Q_ cell; p_1ND_ is the probability that a T_1_ tumor cell is transformed into a non-dividing cell T_ND_ in the blood. The growth of tumor cells represented by the MCM is modelled by Eq. (13). The volume of V_2_ is evaluated in the same way using Eq. (14). The volumes of V_m_, V_Q_, and V_ND_ are expressed by the following equations, since the active tumor is divided into M pieces using Eqs. (15–17).

15 $$ \frac{{dV_{m} }}{dt} = a_{m} V_{m} \left( {1 - \frac{{V_{m} }}{{K_{m} }}} \right) + p_{Qm} V_{Q} - \left( {p_{mQ} + p_{mND} } \right)V_{m} $$

16 $$ \frac{{dV_{Q} }}{dt} = \left( {p_{1Q} V_{1} + p_{2Q} V_{2} + \ldots + p_{mQ} V_{m} } \right) - \left( {p_{Q1} + p_{Q2} + \ldots + p_{Qm} + p_{QND} } \right)V_{Q} $$

17 $$ \frac{{dV_{ND} }}{dt} = p_{1ND} V_{1} + p_{2ND} V_{2} + \ldots + p_{mND} V_{m} + p_{QND} V_{Q} - \eta V_{ND} $$

We used the MCM optimized with M = 2 to simplify the evaluation using this model. The percentages of T_1_, T_2_, and T_Q_ tumor cells in the total tumor volume were defined as 50%, 20%, and 30%, respectively, with good agreement for the measurement value. MCM biological parameters were optimized and matched to measurement values of A549 and NCI-H460 (H460) cells using MATLAB software with the SimBiology toolbox^47,48^.

### The effect of SBRT on the tumor volume using the mathematical model combined with the MKM

The LQM has been used to calculate the cell lethal effect of photon beams^19–21^. In the present study, to more accurately assess the cell lethal effects of SBRT, we evaluated the cell lethal effects using the MKM instead of the LQM.

18 $$ \begin{aligned} S = & e^{{ - \left( {\alpha_{0} + \frac{{y_{D} }}{{\rho \pi r_{d}^{2} }}\beta_{0} } \right)D - F\beta_{0} D^{2} }} \\ & = e^{{ - \alpha_{MKM} D - \beta_{MKM} D^{2} }} \\ \end{aligned} $$

The lethal effects of radiation on the tumors were calculated in the model using the MKM, and Eq. (18) was converted to ODE format (Eq. (19)).

19 $$ \frac{dV}{{dt}} = - \left( {\alpha_{MKM} \dot{D} + 2\beta_{MKM} \dot{D}^{2} } \right)V $$

Here, $\dot{D}$ is the dose rate of radiation, and *V* is the volume of the tumor cells. The combination of tumor cell growth as indicated by the MCM and cell lethality as indicated by the radiological calculation yields the tumor cell volume calculation in SBRT (Eq. (20)).

20 $$ \left\{ \begin{aligned} \frac{{dV_{1} }}{dt} = & a_{1} V_{1} \left( {1 - \frac{{V_{1} }}{{K_{1} }}} \right) + p_{Q1} V_{Q} - \left( {p_{1Q} + p_{1ND} } \right)V_{1} - \left( {\alpha_{MKM1} D + 2\beta_{MKM1} D^{2} } \right)V_{1} \\ \frac{{dV_{2} }}{dt} = & a_{2} V_{2} \left( {1 - \frac{{V_{2} }}{{K_{2} }}} \right) + p_{Q2} V_{Q} - \left( {p_{2Q} + p_{2ND} } \right)V_{2} - \left( {\alpha_{MKM2} D + 2\beta_{MKM2} D^{2} } \right)V_{2} \\ \frac{{dV_{Q} }}{dt} = & \left( {p_{1Q} V_{1} + p_{2Q} V_{2} } \right) - \left( {p_{Q1} + p_{Q2} + p_{QND} } \right)V_{Q} - \left( {\alpha_{MKMQ} D + 2\beta_{MKMQ} D^{2} } \right)V_{Q} \\ \frac{{dV_{ND} }}{dt} = & p_{1ND} V_{1} + p_{2ND} V_{2} + p_{QND} V_{Q} - \eta V_{ND} \\ \end{aligned} \right. $$

Finally, to simulate fractionated irradiation in SBRT, the following equation was defined:

21 $$ V_{1} = \sum\limits_{i = 1}^{N} {\left[ {\int\limits_{{t_{0} }}^{{t_{{{\text{int}} ra}} }} {\left\{ {a_{1} V_{1i} \left( {1 - \frac{{V_{1i} }}{{K_{1} }}} \right) + p_{Q1} V_{Qi} - \left( {p_{1Q} + p_{1ND} } \right)V_{1i} } \right\}} dt - \int\limits_{{t_{0} }}^{{t_{{{\text{int}} er}} }} {\left\{ {\left( {\alpha_{MKM1} D_{i} + 2\beta_{MKM1} D_{i}^{2} } \right)V_{1i} } \right\}} dt} \right]} $$

**Figure 1:**
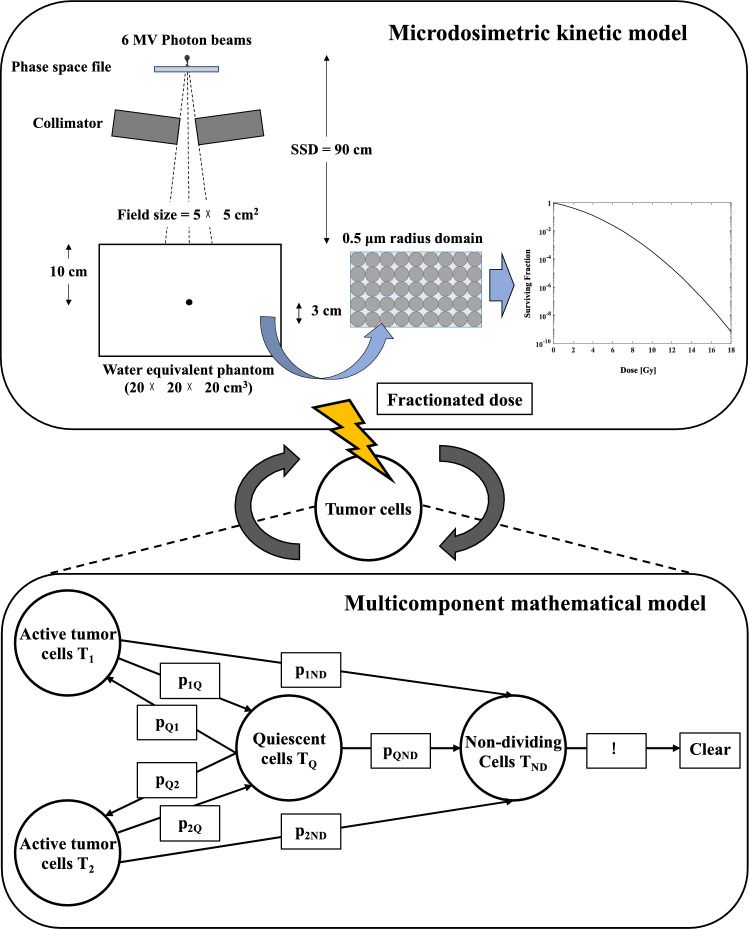
The effect of radiotherapy on tumor volume calculations combining the multicomponent mathematical model (MCM) (lower compartment) representing tumor growth and microdosimetric kinetic model (MKM) (upper compartment) to assess tumor survival.

V_1i_, V_Qi_, and D_i_ are the volume and absorbed dose of the i-th tumor cell at V_1_, V_Q_, and D, respectively. N is the total number of radiotherapy fractions. The prescribed doses in this study were 48 Gy/4 fr and 54 Gy/3 fr, based on the conditions of clinical trials of SBRT for lung cancer^49,50^. The tumor volume in the fractional irradiation of V_2_, V_Q_, and V_ND_ was similarly calculated using Eq. (21). Figure 1 showed the MCM simulates combined with MKM to evaluate the effect of SBRT for tumor growth volume.

The dose rate $\dot{D}$ is 3 Gy/min, and *V* is the volume of the tumor cells, which represents the total tumor volume of V_1_, V_2_, and V_Q_ combined, defined as 500, 1000, and 1500 cm^3^ in this study. The t_inter_ was set to 1 [day] in this study since t_inter_ represents the time interval [days] of the fractionated dose (12 Gy or 18 Gy). The t_intra_ represents the dose delivery time per one fractionated dose. Using the MKM instead of the LQM enabled the calculation of the effect of the dose rate to be taken into account.

In addition to the t_intra_ derived from the relationship between the absorbed dose per fraction and the dose rate, we defined the t_intra_ as 1, 5, 10, 30, and 60 min, and we then evaluated the effect of prolonging the dose delivery time t_intra_ on the tumor volume. The first irradiation was defined as Time = 0 [day], the third irradiation, the final irradiation of 54 Gy/3 fr, was defined as Time = 2 [day], and the fourth irradiation, the final irradiation of 48 Gy/4 fr, was defined as Time = 3 [day]. We used the ratio of the tumor volume at 1 day after the end of the irradiation to the initial tumor volume to define the radiation effectiveness value (REV) in order to evaluate the effect of SBRT on the two NSCLC cell lines, A549 and H460.

## Results

### Comparison of the measured tumor volume and calculated tumor volume by MCM in A549 and H460 NSCLC cells

**Figure 2:**
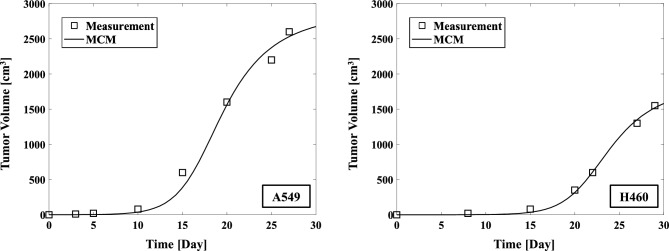
Comparison of the tumor growth volume measurements of A549 (*left*) and H460 (*right*) NSCLC cells with the values calculated by the MCM.

Figure 2 shows the measured values of A549 and H460 and the tumor growth volume calculated using the MCM. The parameters of the MCM for tumor growth volume are given in Table 1. The calculated results of the MCM for tumor volume evolution in time showed good agreement with the measurement values with both cell lines.

**Table 1:** The tumor growth volume calculation parameters for the MCM using A549 and NCI-H460 non-small cell lung cancer cells.

Parameter	A549	H460
$a_{1} \left( {Day^{ - 1} } \right)$	0.86	0.76
$a_{2} \left( {Day^{ - 1} } \right)$	0.50	0.50
$K_{1} (mm^{3} )$	1397	757
$K_{2} (mm^{3} )$	1174	1199
$p_{Q1} \left( {Day^{ - 1} } \right)$	0.1	0.1
$p_{1Q} \left( {Day^{ - 1} } \right)$	0.2	0.2
$p_{1ND} \left( {Day^{ - 1} } \right)$	0.2	0.2
$p_{Q2} \left( {Day^{ - 1} } \right)$	0.1	0.1
$p_{2Q} \left( {Day^{ - 1} } \right)$	0.2	0.2
$p_{2ND} \left( {Day^{ - 1} } \right)$	0.2	0.2
$p_{QND} \left( {Day^{ - 1} } \right)$	0.09	0.09
$\eta \left( {Day^{ - 1} } \right)$	0.4	0.4

### Comparison of the surviving fraction (SF) for the A549 and H460 cells predicted by the LQM and MKM models

**Figure 3:**
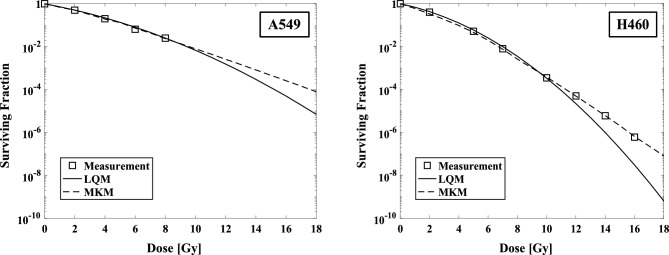
The surviving fraction (SF) values calculated by the LQM and the MKM models compared to the measured values, presented for A549 (left) and H460 (right).

The comparison of calculated SF by LQM and MKM for the measured tumor SF from irradiation of A549 and H460 cells were shown (Fig. 3). In the high-dose region (> 12 Gy), the LQM underestimate the SF compared to measured values, whereas the MKM calculations show good agreement in the high-dose region. The parameters of the MKM for the tumor SF calculation are given in Table 2. The calculated cell viability with LQM and MKM for the measured cell viability with irradiation in A549 and H460 cells are shown (Fig. 3).

**Table 2:** The MKM parameters used to calculate the tumor surviving fraction for A549 and H460 non-small cell lung cancer cells.

Parameter	A549	H460
$\alpha_{1} \left( {Gy^{ - 1} } \right)$	0.19	0.21
$\alpha_{2} \left( {Gy^{ - 1} } \right)$	0.37	0.16
$\alpha_{Q} \left( {Gy^{ - 1} } \right)$	0.30	0.30
$\beta_{1} \left( {Gy^{ - 2} } \right)$	0.06	0.07
$\beta_{2} \left( {Gy^{ - 2} } \right)$	0.02	0.03
$\beta_{Q}$ $\left( {Gy^{ - 2} } \right)$	0.15	0.15
$\left( {a + c} \right)\left( {h^{ - 1} } \right)$	2.10	1.51
$y_{D} \left( {keV/mm} \right)$	2.38 ± 0.01	2.38 ± 0.01
$\dot{D} \left( {Gy/minute} \right)$	3.0	3.0
$t_{intra} \left( {minute} \right)$	4.0 (12 Gy), 6.0 (18 Gy)	4.0 (12 Gy), 6.0 (18 Gy)
$t_{inter} \left( {Day} \right)$	1.0	1.0

### The evaluation of effect of SBRT on tumor volumes using the MCM combined with the LQM and the MKM

**Figure 4:**
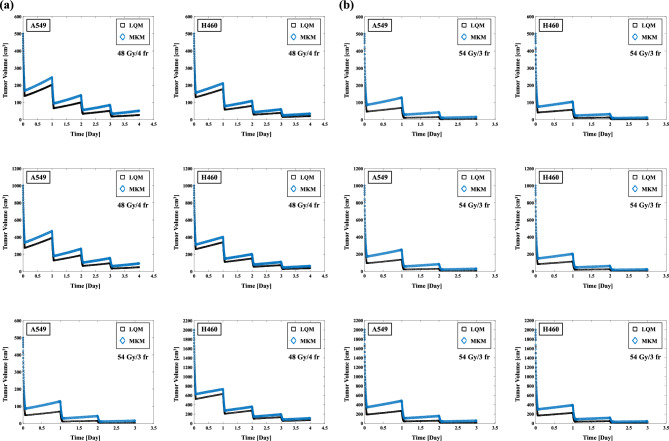
(**a**) Effect on each initial tumor volume (500, 1000, 2000 cm^3^) in 48 Gy/4 fr irradiation of A549 and H460 non-small cell lung cancer cells (left two columns). (**b**) Effect on each initial tumor volume (500, 1000, 2000 cm^3^) in 54 Gy/3 fr irradiation of A549 and H460 cells (right two columns).

A combined MCM and LQM or MKM model was used to evaluate the impact of SBRT on the tumor volume. The effects of the uses of the LQM and MKM on the tumor volume in SBRT are illustrated in Fig. 4a,b. The REVs for A549 cells on 500, 1000, 2000 cm^3^ using the LQM were 94.64%, 94.98%, and 95.53%, respectively (Table 3) with SBRT at 48 Gy/4 fr. The REVs using the MKM were 89.73%, 90.66%, and 92.05%, respectively. The REVs for A549 cells on 500, 1000, 2000 cm^3^ using the LQM were 99.21%, 99.22%, and 99.25%, respectively (Table 3) in 54 Gy/3 fr SBRT. The REVs using the MKM were 96.97%, 97.06%, and 97.23%, respectively. The REVs obtained with the MKM were lower than those obtained with the LQM, and the difference in REVs between the LQM and the MKM resulted in a smaller difference when the SBRT dose was 54 Gy/3 fr. In addition, the H460 cells have higher REV values than A549 cells in each of the cases (Table 3).

**Table 3:** The effect of SBRT (48 Gy/4 fr and 54 Gy/3 fr) on the REVs for A549 and H460 non-small cell lung cancer cells using the MCM combined with the LQM and the MKM.

NSCLC cell line	Model, dose/fx	REV, (%)		
	Volume, (cm^3^)	500	1000	2000
A549	LQM (48 Gy/4 fr)	94.64	94.98	95.53
	MKM (48 Gy/4 fr)	89.73	90.66	92.05
H460	LQM (48 Gy/4 fr)	95.89	96.13	96.52
	MKM (48 Gy/4 fr)	93.16	93.69	94.51
A549	LQM (54 Gy/3 fr)	99.21	99.22	99.25
	MKM (54 Gy/3 fr)	96.97	97.06	97.23
H460	LQM (54 Gy/3 fr)	99.34	99.35	99.37
	MKM (54 Gy/3 fr)	97.73	97.79	97.91

### The impact of varying the V_1_, V_2_ and V_Q_ ratio on tumor volume in SBRT

**Figure 5:**
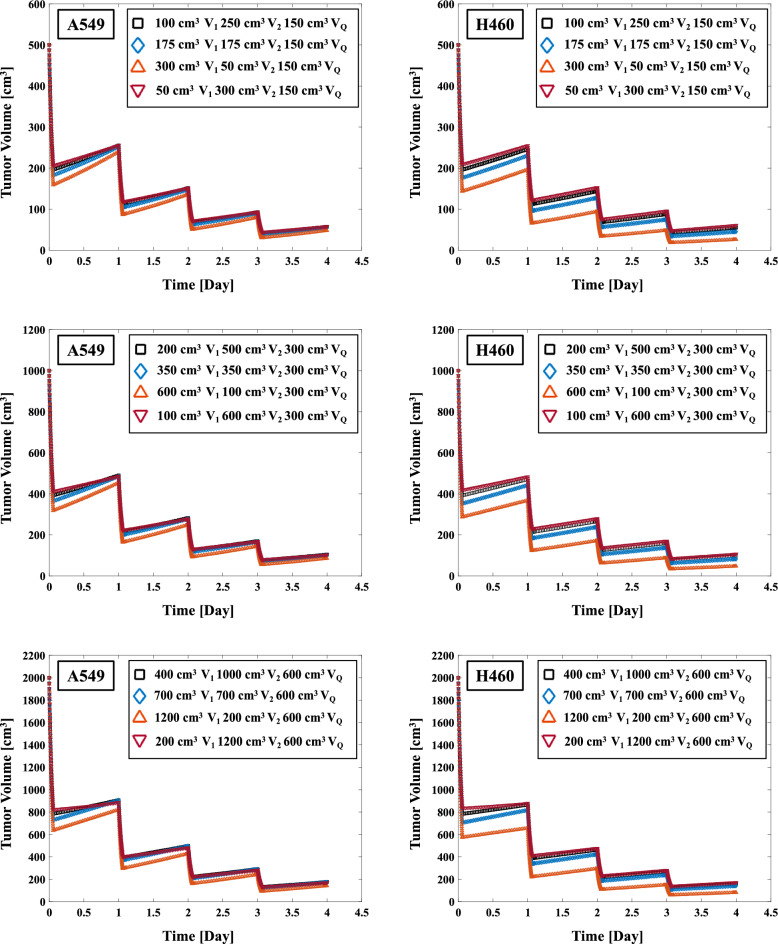
Effect of varying the V_1_ and V_2_ ratio for total tumor volume on the REVs in 48 Gy/4 fr irradiation of A549 and H460 non-small cell lung cancer (NSCLC) cells.

**Figure 6:**
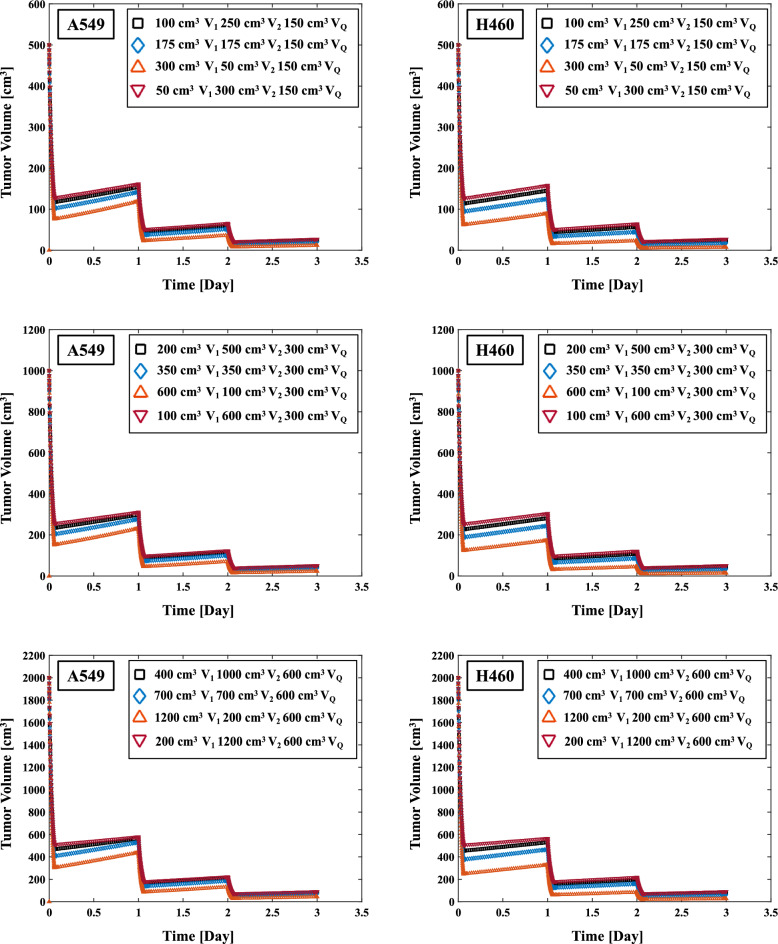
Effect of varying the V_1_ and V_2_ ratio on the REVs in 54 Gy/3 fr irradiation of A549 and H460 NSCLC cells.

To determine its effect on the REVs in SBRT, we varied the ratios of V_1_, V_2_, and V_Q_ tumor cells for the total tumor volume. Figures 5 and 6 depicts the results of the varying the ratio of V_1_ and V_2_ on the REVs. The percentage of V_Q_ in the total tumor volume was fixed (30%, 500 cm^3^, 1000 cm^3^, and 2000 cm^3^) and we varied the ratio of V_1_–V_2_ at 10%, 20%, 35%, and 60% to evaluate the REVs. Table 4 summarizes the effects of varying the V_1_, V_2_, and V_Q_ ratios for the REVs in SBRT (48 Gy/4 fr and 54 Gy/3 fr). The REVs on 500 cm^3^ initial tumor volume in 48 Gy/4 fr of A549 cells with 20% V_1_ (100 cm^3^) 50% V_2_ (250 cm^3^) 30% V_Q_ (150 cm^3^), 35% V_1_ (100 cm^3^) 35% V_2_ (100 cm^3^) 30% V_Q_ (800 cm^3^), and 60% V_1_ (300 cm^3^) 10% V_2_ (50 cm^3^) 30% V_Q_ (150 cm^3^) were 88.53%, 88.98%, and 90.41%, respectively (Table 4). The corresponding values with 54 Gy/3 fr were 95.14%, 96.03%, and 97.63%, respectively. In the 48 Gy/4 fr and 54 Gy/3 fr SBRT, the highest REVs were observed for both A549 and H460 cells with a larger ratio of V_1_ in the total tumor volume on 500, 1000, and 2000 cm^3^. In addition, the tendency for an increase in the ratio of V_1_ to increase the REVs were greater for the 54 Gy/3 fr compared to the 48 Gy/4 fr (Table 4).

**Table 4:** The impact of varying the V_1_, V_2_, and V_Q_ ratio on the REVs in SBRT (48 Gy/4 fr and 54 Gy/3 fr) for non-small cell lung cancer cells (A549 and H460).

NSCLC cell line	Model, dose/fx	REV, (%)		
	Volume, (cm^3^)	500	1000	2000
A549	20% V_1_ 50% V_2_ 30% V_Q_ (48 Gy/4 fr)	88.53	89.60	91.20
	35% V_1_ 35% V_2_ 30% V_Q_ (48 Gy/4 fr)	88.98	89.89	91.32
	60% V_1_ 10% V_2_ 30% V_Q_ (48 Gy/4 fr)	90.41	91.45	92.91
	10% V_1_ 60% V_2_ 30% V_Q_ (48 Gy/4 fr)	88.41	89.67	91.47
	10% V_1_ 10% V_2_ 80% V_Q_ (48 Gy/4 fr)	85.71	87.21	89.44
	20% V_1_ 20% V_2_ 60% V_Q_ (48 Gy/4 fr)	86.35	87.73	89.79
H460	20% V_1_ 50% V_2_ 30% V_Q_ (48 Gy/4 fr)	89.00	90.16	91.87
	35% V_1_ 35% V_2_ 30% V_Q_ (48 Gy/4 fr)	90.95	91.72	92.93
	60% V_1_ 10% V_2_ 30% V_Q_ (48 Gy/4 fr)	94.80	95.27	95.93
	10% V_1_ 60% V_2_ 30% V_Q_ (48 Gy/4 fr)	87.84	89.33	91.41
	10% V_1_ 10% V_2_ 80% V_Q_ (48 Gy/4 fr)	88.26	89.54	91.41
	20% V_1_ 20% V_2_ 60% V_Q_ (48 Gy/4 fr)	88.78	89.96	91.69
A549	20% V_1_ 50% V_2_ 30% V_Q_ (54 Gy/3 fr)	95.14	95.40	95.84
	35% V_1_ 35% V_2_ 30% V_Q_ (54 Gy/3 fr)	96.03	96.18	96.46
	60% V_1_ 10% V_2_ 30% V_Q_ (54 Gy/3 fr)	97.63	97.71	97.85
	10% V_1_ 60% V_2_ 30% V_Q_ (54 Gy/3 fr)	94.58	94.93	95.50
	10% V_1_ 10% V_2_ 80% V_Q_ (54 Gy/3 fr)	94.70	94.97	95.44
	20% V_1_ 20% V_2_ 60% V_Q_ (54 Gy/3 fr)	94.96	95.21	95.64
H460	20% V_1_ 50% V_2_ 30% V_Q_ (54 Gy/3 fr)	95.31	95.57	96.02
	35% V_1_ 35% V_2_ 30% V_Q_ (54 Gy/3 fr)	96.49	96.64	96.89
	60% V_1_ 10% V_2_ 30% V_Q_ (54 Gy/3 fr)	98.58	98.61	98.68
	10% V_1_ 60% V_2_ 30% V_Q_ (54 Gy/3 fr)	94.55	94.91	95.51
	10% V_1_ 10% V_2_ 80% V_Q_ (54 Gy/3 fr)	95.33	95.58	96.01
	20% V_1_ 20% V_2_ 60% V_Q_ (54 Gy/3 fr)	95.56	95.78	96.17

**Figure 7:**
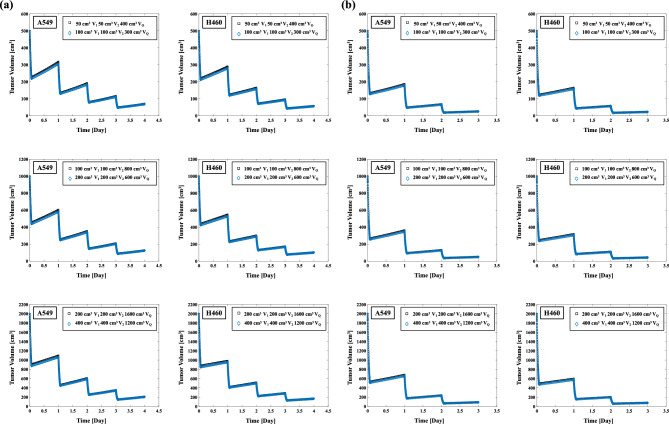
(**a**) Effect of the V_Q_ ratio on the total tumor volume in 48 Gy/4 fr irradiation of A549 and H460 non-small cell lung cancer cells (left two columns). **(b**) Effect of the V_Q_ ratio on the total tumor volume in 54 Gy/3 fr irradiation of A549 and H460 cells (right two columns).

Figure 7a,b illustrates the effect on the REVs of changing the ratio of V_Q_. The REV in 48 Gy/4 fr of A549 with 10% V_1_ (50 cm^3^) 10% V_2_ (50 cm^3^) 80% V_Q_ (400 cm^3^) on 500 cm^3^ initial tumor volume was 85.71% (Table 4). The corresponding values with 20% V_1_ (100 cm^3^) 20% V_2_ (100 cm^3^) 60% V_Q_ (300 cm^3^) on 500 cm^3^ initial tumor volume was 86.35% (Table 4). The higher the V_Q_ ratio resulted in no significant effect on REV values. The effect of the V_Q_ ratio on the REV was the same for the H460 cells and 54 Gy/3 fr.

### The effect of the dose delivery time per one fractionated dose t_intra_ for REV in SBRT

**Figure 8:**
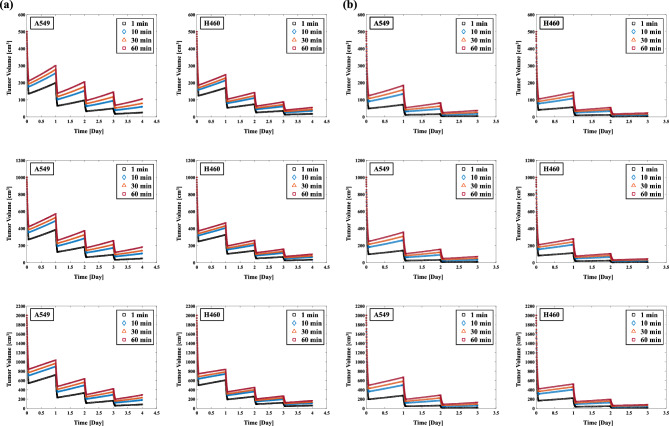
(**a**) Effect of the dose delivery time t_intra_ on the REVs in 48 Gy/4 fr irradiation of A549 and H460 non-small cell lung cancer cells (*left two columns*). (**b**) The influence of the dose delivery time t_intra_ on the REVs in 54 Gy/3 fr irradiation of A549 and H460 cells (*right two columns*).

Figure 8a shows the effect of changing the dose delivery time per one fractionated dose t_intra_ on the REV in A549 and H460 cells irradiated at 48 Gy/4 fr. The REVs of the A549 cells with 1 and 60 min t_intra_ were 94.93% and 79.00% for 500 cm^3^, respectively (Table 5), and 95.75% and 85.46% for 2000 cm^3^, respectively. The REVs of the H460 cells with 1 and 60 min t_intra_ were 96.39% and 88.80% for 500 cm^3^, respectively, and 96.91% and 91.59% for 2000 cm^3^, respectively. Figure 8b illustrates the effect of extending the dose delivery time per one fractionated dose t_intra_ on the REVs for A549 and H460 cells irradiated at 54 Gy/3 fr. The REVs of the A549 cells with 1 and 60 min t_intra_ were 99.15% and 92.37% for 500 cm^3^, respectively (Table 5), and 99.19% and 93.49% for 2000 cm^3^, respectively. The REVs of the H460 cells with 1 and 60 min t_intra_ were 99.35% and 95.25% for 500 cm^3^, respectively, and 99.38% and 95.81% for 2000 cm^3^, respectively. The REVs were significantly decreased with the increase in the dose delivery time t_intra_; in addition, the smaller the tumor volume, the greater the effect of increasing the t_intra_ was. The effect of the extended irradiation time on the REV reduction was smaller for 54 Gy/3 fr compared to 48 Gy/4 fr.

**Table 5:** Effect on varying the dose delivery time t_intra_ on the REV in the tumor volume of A549 and H460 non-small cell lung cancer cells in SBRT.

NSCLC cell line	Model, dose/fx	REV, (%)		
	Volume, (cm^3^)	500	1000	2000
A549	1 min (48 Gy/4 fr)	94.93	95.24	95.75
	10 min (48 Gy/4 fr)	88.10	89.27	90.98
	30 min (48 Gy/4 fr)	84.25	86.05	88.55
	60 min (48 Gy/4 fr)	79.00	81.79	85.46
H460	1 min (48 Gy/4 fr)	96.39	96.58	96.91
	10 min (48 Gy/4 fr)	92.87	93.44	94.31
	30 min (48 Gy/4 fr)	91.36	92.13	93.27
	60 min (48 Gy/4 fr)	88.80	89.95	91.59
A549	1 min (54 Gy/3 fr)	99.15	99.16	99.19
	10 min (54 Gy/3 fr)	96.59	96.70	96.91
	30 min (54 Gy/3 fr)	94.87	95.09	95.47
	60 min (54 Gy/3 fr)	92.37	92.79	93.49
H460	1 min (54 Gy/3 fr)	99.35	99.36	99.38
	10 min (54 Gy/3 fr)	97.51	97.59	97.72
	30 min (54 Gy/3 fr)	96.58	96.70	96.91
	60 min (54 Gy/3 fr)	95.25	95.45	95.81

## Discussion

In this study, we evaluated the effect of two different prescribed doses of SBRT on NSCLC cells by combining an MCM, which calculates tumor growth, and the LQM or the MKM, which calculate cell lethality from radiotherapy. As suggested in earlier studies^26–31^, there is a possibility of overestimating the SF in the high-dose range > 10 Gy when the LQM is used, suggesting the possibility of good SF prediction by using the MKM as reported (Fig. 3)^37,38,51,52^. In the present study therefore, the REVs for A549 and H460 cells had larger values for the LQM compared to the MKM: up to 4.9% and 2.7% higher at 48 Gy/4fr and 2.2% and 1.6% higher at 54 Gy/3fr (Table 3). The reason for the larger difference between the LQM and MKM REV in the A549 cells is thought to be that the difference in calculated SFs between the LQM and the MKM in the A549 cells was larger than that in the H460 cells (Fig. 3). In the case of 54 Gy/3 fr, the fractionated dose is larger than that of 48 Gy/4fr, which might explain the smaller difference in REV reduction between the LQM and MKM models, as shown in Table 3. A mathematical model combined with an MKM based on ordinary differential equations could thus be used to more accurately calculate the tumor volume for NSCLC in SBRT.

Figures 5 and 6 showed the effect of varying the ratio of V_1_, V_2_ on the REV. The difference in the REVs at 48 Gy/4 fr was up to 4.7% for A549 cells and up to 6.5% for H460 cells at an initial tumor volume of 500 cm^3^ (Table 4), and the maximum was 3.1% for A549 cells and 4.0% for H460 cells at an initial tumor volume of 500 cm^3^ at 54 Gy/3 fr. The greater the percentage of V_1_ in the total tumor volume, the lower the REV was. The reason for this might be that V_1_ represents the volume of active tumor T_1_, which has a smaller α/β value compared to active tumor T_2_ (Table 1). In radiobiology, the effect is higher for a larger fractionated dose when the α/β ratio is small^53^.

The change in the percentage of quiescent cells T_Q_ (V_Q_) also affected the REV (Table 4). The low radiosensitivity of quiescent cells compared to active tumor cells^54^. The advanced tumors have quiescent cells that grow slowly, and that the growth of human solid tumors depends not only on rapidly growing cancer cells, but also on their continued production^55^. Our comparison of the impact of V_1_ and V_2_ of active tumors and that of V_Q_ of quiescent tumors as a ratio of the total tumor volume on the REV revealed that V_1_ and V_2_ had a greater impact on the REV. An estimated assessment of the ratio of active tumor to total tumor volume may therefore be needed for determining the REV; such an estimation is a task for a future study.

The flow cytometry was used to analyze the cell cycle distribution for the A549 and H460 lung cancer cell lines and observed that (1) the active tumors are highly dependent on the cell cycle, and (2) the rate of active tumors varies between tumor cell lines^56^. In addition, the tumor α/β values for the surviving fraction have been reported to vary even for the same type of lung cancer^57^. In the future, in order to optimize the parameters of the model, it will be necessary to collect experimental data and clinical data and optimize parameters such as V_1_, V_2_, V_Q_, and α/β.

There are several reports regarding the effect of a prolonged dose-delivery time (t_intra_) on tumor cell survival, and a prolonged dose-delivery time was reported to decrease the effect on tumors^37,40,41,58^. The delivered biologically effective dose (BED) levels were calculated due to alterations in the SLDR, and a loss of BED was observed on 13 Gy with the dose-delivery time of 35 min compared to the acute-exposure approach. The effect of a prolonged dose delivery time using the MKM on tumors showed an approximate 6% decrease in relative biological effectiveness at an irradiation time of 60 min^40^. In the present study, a tumor volume of 500 cc at 48 Gy/4 fr and an irradiation time of 60 min showed the greatest REV reduction at 15.9% in A549 cells (Table 5). On the other hand, H460 cells showed a 7.6% REV reduction under the same conditions. The greater impact of a time extension on the REV for A549 cells compared to H460 cells might be due to the faster DNA repair time for A549 cells and the greater impact of SLDR (Table 2).

We suspect that the reason for the smaller REV reductions in both A549 and H460 cells at 54 Gy/3 fr compared to 48 Gy/4 fr is that the cell lethal effect of the larger single fractionated dose is less affected by the SLDR effect of the longer dose-delivery time. Considering the results of the single irradiation in other studies that evaluated a prolonged dose-delivery time together with the results of the fractionated dose used in the present study, our results showed a decrease in the REV, similar to the previous studies. Few investigations have taken into account the dose-delivery time of each fraction in a fractionated dose and calculated the effect of the fractionated dose on the tumor. Our present findings suggest that (1) the dose-delivery time might have an effect on tumors even in a fractionated dose, and (2) it might be necessary to attempt to increase the dose-delivery time as quickly as possible.

This study has two limitations to address. The first limitation is our evaluation of the REV by SBRT using ODE to estimate tumor growth and the cell lethality calculation model MKM, without considering changes in the tumor cell environment after irradiation. The high single radiation doses delivered, such as SBRT, may induce elevated and possibly persistent tumor hypoxia in NSCLC tumors^59^. Changes in tumor oxygenation after irradiation may alter the tumor response to radiation therapy (may impact effectiveness of radiation therapy); this requires further investigation^60,61^. The second study limitation is that we set 1 day after irradiation as the time point for the evaluation of the REV. For accurate REV derivation, it will be necessary to evaluate the effect on tumors with using more time evaluation points, as in clinical trials^8,46,47^.

## Conclusions

We evaluated the tumor volume considering a large fractionated dose and the dose-delivery time by combining the MKM with a mathematical model of tumor growth using ODEs in lung SBRT for non-small cell lung cancer. Our results demonstrated that the ratio of active tumor to the total tumor volume and the dose-delivery time both affect the tumor volume in SBRT.

## Data Availability

The data that support the findings of this study are available from corresponding author but restrictions apply to the availability of these data, which were used under license for the current study, and so are not publicly available. Data are however available from the authors upon reasonable request and with permission of corresponding author.
